# SPE-8, a protein-tyrosine kinase, localizes to the spermatid cell membrane through interaction with other members of the SPE-8 group spermatid activation signaling pathway in *C. elegans*

**DOI:** 10.1186/1471-2156-15-83

**Published:** 2014-07-14

**Authors:** Paul J Muhlrad, Jessica N Clark, Ubaydah Nasri, Nicholas G Sullivan, Craig W LaMunyon

**Affiliations:** 1 Department of Molecular and Cellular Biology, University of Arizona, 1007 Lowell St., Tucson, AZ 85721, USA; 2 Department of Molecular, Cellular and Developmental Biology, Currently, University of Colorado Boulder, 347 UCB, Boulder, CO 80309, USA; 3 Department of Biological Science, California State Polytechnic University, 3801 W. Temple Ave, Pomona, CA 91768, USA

**Keywords:** Caenorhabditis elegans, Spermatogenesis, Sperm activation, spe-8, Signal transduction

## Abstract

**Background:**

The SPE-8 group gene products transduce the signal for spermatid activation initiated by extracellular zinc in *C. elegans*. Mutations in the *spe-8* group genes result in hermaphrodite-derived spermatids that cannot activate to crawling spermatozoa, although spermatids from mutant males activate through a pathway induced by extracellular TRY-5 protease present in male seminal fluid.

**Results:**

Here, we identify SPE-8 as a member of a large family of sperm-expressed non-receptor-like protein-tyrosine kinases. A rescuing SPE-8::GFP translational fusion reporter localizes to the plasma membrane in all spermatogenic cells from the primary spermatocyte stage through spermatids. Once spermatids become activated to spermatozoa, the reporter moves from the plasma membrane to the cytoplasm. Mutations in the *spe-8* group genes *spe-12, spe-19,* and *spe-27* disrupt localization of the reporter to the plasma membrane*,* while localization appears near normal in a *spe-29* mutant background.

**Conclusions:**

These results suggest that the SPE-8 group proteins form a functional complex localized at the plasma membrane, and that SPE-8 is correctly positioned only when all members of the SPE-8 group are present, with the possible exception of SPE-29. Further, SPE-8 is released from the membrane when the activation signal is transduced into the spermatid.

## Background

The timing of sperm activation requires precise regulation. Activating too early wastes stored resources, and delayed activation put the sperm at a competitive disadvantage. Sperm activation, and spermatogenesis as a whole, have emerged as an important model system in the hermaphroditic nematode *C. elegans*[1]. Activation, also called spermiogenesis in *C. elegans,* is the final developmental transition in spermatogenesis when quiescent spherical spermatids undergo wholesale cellular reorganization, extend pseudopods, and begin to crawl. Two signaling molecules induce spermiogenesis: extracellular zinc [2] and the extracellular protease TRY-5 [3]. Both pathways are employed for male sperm activation, but only the zinc pathway is utilized in hermaphrodites for self-sperm activation [2]. The zinc signal is transduced into the spermatid via the products of at least five genes (*spe-8, spe-12, spe-19, spe-27,* and *spe-29*) named the *spe-8* pathway for the first of the genes discovered [4]. Loss-of-function mutations in any of these genes result in an identical phenotype: hermaphrodites are self-sterile because their spermatids do not activate, while male mutants are fertile. Mutant hermaphrodites become self-fertile when inseminated by males because their self-spermatids are activated by TRY-5 in the seminal fluid. Given that *spe-12, spe-19,* and *spe-29* encode membrane proteins [5-7], it has been proposed that a complex of the SPE-8 group proteins forms at the cell membrane to transduce the zinc-derived signal into the cell. Although *spe-8* was the first gene of this group to be named, the molecular identity of *spe-8* has not been published. Here, we report the identification of the *spe-8* coding sequence, show that it encodes a member of a large family of sperm-expressed protein tyrosine kinases, and demonstrate that its gene product localizes to the membrane in a manner dependent upon the presence of the other *spe-8* group gene products.

## Results

### Identification of the *spe-8* coding sequence

**Figure 1:**
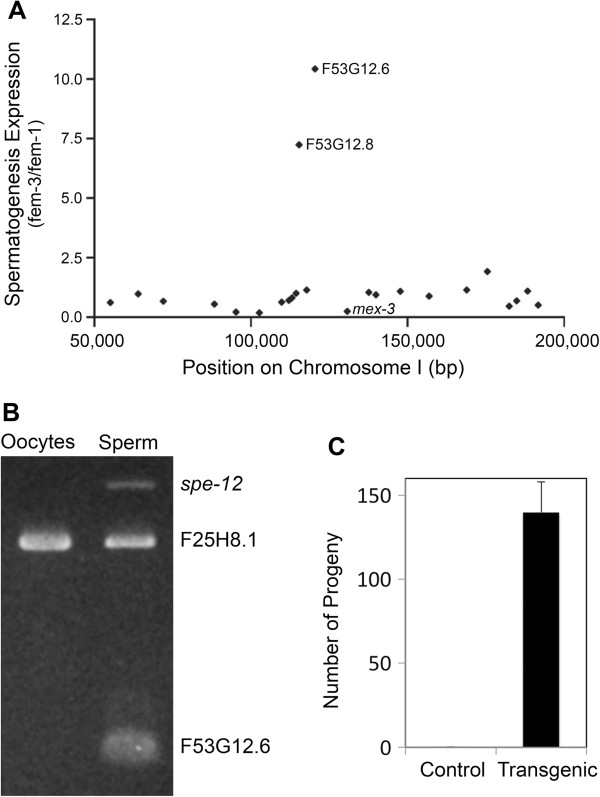
**Identification of the spe-8 coding sequence. A**. The graph shows all the predicted genes on the far left end of Chromosome I, where *spe-8* and *mex-3* are known to be very close to one another. The Y axis plots the degree of sperm-specific expression measured as *fem-3(q23)/fem-l(hcl7)* microarray signal intensity [9,10]. Two genes in the region, F53Gl2.6 and F53Gl2.8, appear spermatogenesis-specific. F53Gl2.8 had been previously eliminated as a candidate based on other results (Jeremy Nance, personal communication). **B**. Differential PCR analysis was used to confirn the sperm-specific expression of F53G12.6. PCR products were amplified from cDNA libraries derived either from *fem-1(hcl7ts)* hermaphrodites which produce only oocytes, or from *fem-3(q23ts)* hermaphrodites which produce only sperm. The PCR reactions were multiplexed for three possible products: (i) a 169 bp product from F53G12.6 cDNA, (ii) a 526 bp product from F25H8.1, a somatic gene included as a non-germline-specific control, and (iii) a 714 bp product from *spe-12*, included as a sperm-specific control. Both F53G12.6 and *spe-12* products appear sperm specific by their appearance only in the *fem-3* library, whereas the product from the somatic gene F25H8.1 was found in both libraries as expected. (PCR primers are given in Additional file 1). **C**. The results of transformation-rescue of *spe-8(hc50)* with wild-type PCR product containing F53G12.6 and its flanking sequence. Transformant worms produced a mean of 139.8 progeny (n = 12; SEM = 18.0) compared to a mean of 0.1 progeny (n = 15; SEM = 0.1) produced by their non-transformed siblings, indicating that F53G12.6 is *spe-8.*

**Figure 2:**
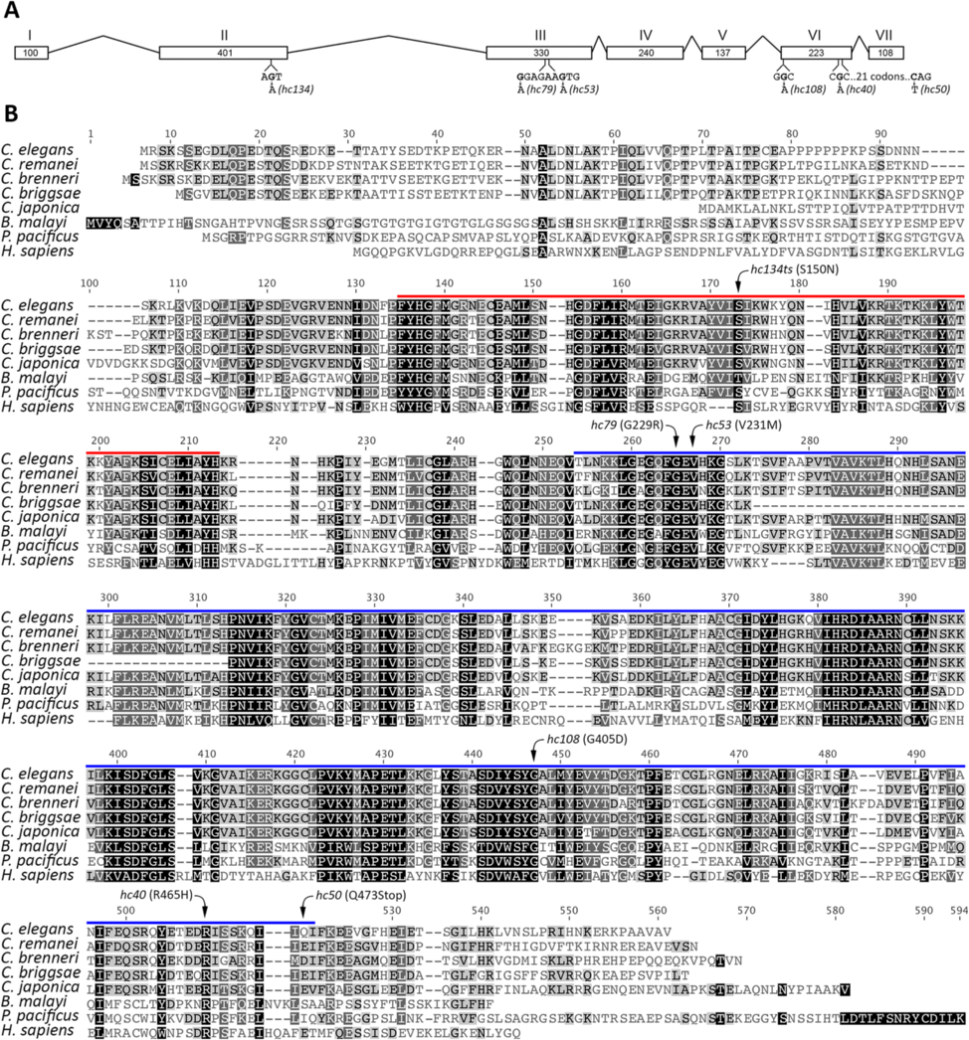
***spe-8 *exonic structure and sequence alignment of its protein product.** The exonic structure shown in A is supported by numerous EST sequences curated by WormBase (http://www.wormbase.org). The locations of mutations in the *spe-8* gene were mapped onto **(A)** the exon structure and **(B)** a multiple alignment of SPE-8 from *C. elegans* and the top BLAST matches from other nematodes. The nucleotide alterations to codons are shown in A, and the amino acid changes are shown in B. Included in the alignment is the human c-Abl Tyrosine Kinase from structural analysis of the protein (Figure 3). The location of the SH2 domain is indicated by the red line, and the kinase domain is indicated by the blue line.

Previous mapping placed *spe-8* to the left end of Chromosome I, approximately 8.7 cM to the left of *lin-17*[4]. Subsequently, *spe-8* was positioned extremely close (inseparable) to *mex-3*[8] (Figure 1A). Because essentially all *spe* and *fer* genes in *C. elegans* exhibit sperm-specific expression [9,10], we reasoned that *spe-8* expression is also specific to spermatogenesis. We surveyed microarray data comparing transcript abundance in hermaphrodites making only sperm [*fem-3(q23gf)*] with hermaphrodites producing only oocytes [*fem-1(hc17ts)*] [9,10]. Of the predicted genes near *mex-3*, only two genes, F53G12.6 and F53G12.8, had a *fem-*3/*fem-1* ratio >5, indicating sperm-specific expression (Figure 1A). F53G12.8 had already been eliminated as a candidate based on other results (Jeremy Nance, personal communication). We confirmed the sperm-specific expression of F53G12.6 by successful amplification of a PCR product specific from this gene in a *fem-3* cDNA library but not in a *fem-1* cDNA library (Figure 1B). Furthermore, a PCR product containing F53G12.6 plus its flanking DNA rescued *spe-8(hc50)* mutants (Figure 1C). Finally, we identified mutations that altered the F53G12.6 coding sequence in each of the *spe-8* mutant strains (Figure 2). Taken together, these data indicate that F53G12.6 is the *spe-8* gene.

### SPE-8 is a member of a family of *C. elegans* sperm proteins with non-receptor protein-tyrosine kinase and SH2 domains

**Figure 3:**
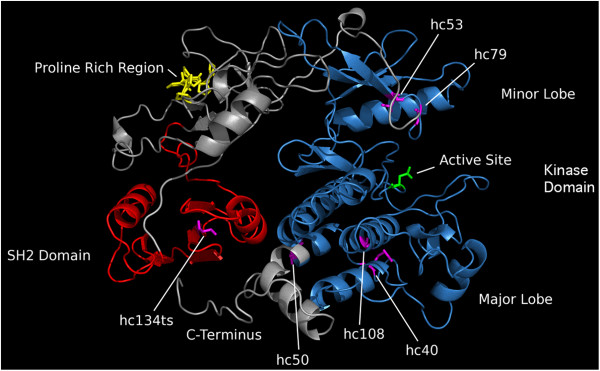
**Predicted structural model of SPE-8.** The model was predicted by TM-align [11] using iTasser [12], and is based on the crystal structure of the human c-Abl Tyrosine Kinase protein. The highly significant structural predictions for the SH2 and kinase domains are indicated by red and blue labeling, respectively. The structures of the N- and C-terminal domains were predicted by iTasser without known protein models and are labeled in gray. The amino acids affected by the *spe-8* mutations are indicated in stick form and labeled purple. Also shown are the predicted kinase active site in green and a proline rich domain in gold.

The SPE-8 protein contains two domains, the first of which is an SH2 domain (Figure 2B, red line, and Figure 3). SH2 (Src homology 2) is a large family of protein domains that bind particular phosphorylated tyrosine residues on other proteins, and are key regulators of tyrosine phosphorylation signaling cascades [13]. Closely following the SH2 domain is a non-receptor protein-tyrosine kinase catalytic domain (Figure 2B, blue line, and Figure 3). SPE-8 is a member of the Fes/Fer protein-tyrosine kinase family [14,15]. Alignment of the SPE-8 protein sequence with those of the best matching homologs from a BLASTP search against the nonredundant set of protein databases shows a high degree of conservation and the common presence of the N-terminal SH2 domain followed by a protein-tyrosine kinase domain, even among distantly related nematodes (Figure 2).

**Figure 4:**
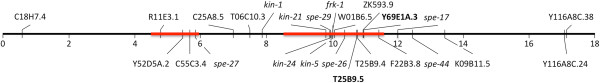
**The clustered arrangement of the sperm-expressed SPE-8 paralogs on Chromosome IV.** The locations of the genes are based upon their nucleotide position, and those above the line are encoded on the positive strand, whereas those below the line are on the negative strand. Known *spe* genes are also shown. Chromosomal regions highlighted in red indicate known sperm gene clusters [17], and bold font indicates the two sperm genes on Chromosome IV with a sterile knockout phenotype.

A BLASTP of SPE-8 against *C. elegans* proteins returns a long list of paralogs (Additional file 2). For the 33 with a match significance ≤ 10^−30^, we examined two sources for information on sperm expression. One was the *fem-3/fem-1* ratios from microarray data [9,10], and the second was an analysis of the sperm transcriptome via RNA deep sequencing of sperm purified from *him-5(e1490)* males [16]. Twenty-nine of the proteins are upregulated in sperm in at least one of the two studies (Additional file 2). Such a large set of sperm-specific SPE-8 paralogs is not surprising, since spermatogenesis in *C. elegans* involves an over-abundance of kinases [9,16]. All of the 29 sperm-expressed paralogs have the N-terminal SH2 domain followed by the kinase domain. Further, the majority (20) are located on Chromosome IV in a non-random distribution, with some located in two known sperm gene clusters [17] while others lie outside the clusters (Figure 4). The N-terminal ~100 residues of SPE-8 define the only region of the sequence that is unique among the sperm-specific paralogs, but the N-terminal domain matches that of all but one of the nematode SPE-8 homologs (Figure 2). Thus, the N-terminal domain likely provides functional specificity for SPE-8. Finally, each of the *Caenorhabditis* congeners shown in Figure 2B contains a large number of paralogs to its own SPE-8 homolog (data not shown).

We modeled the SPE-8 structure using the I-Tasser Protein Structure and Function Prediction Server [12]. The most significant structural alignments were to the human proteins c-Abl Tyrosine Kinase (Accession: 2FO0) and c-Src Tyrosine Kinase (Accession: 1FMK). While both proteins also included an SH3 domain very close to the N-Terminus, both proteins possess the SH2 and kinase domains and have crystal structures available [18,19]. The c-Abl Tyrosine Kinase crystal structure was used for modeling SPE-8 with TM-align [11] because it had the most significant structural match (TM-score = 0.866; Figures 2 and 3). However, SPE-8 has N-terminal and C-terminal ends that were not predicted by c-Abl Tyrosine Kinase structure. We found no significant alignments or predictions for the N-terminal domain (AA’s 1–104) considered alone; however, it contains a stretch of seven sequential prolines that may mediate protein-protein interactions [20].

The effects of the various mutations in the *spe-8* gene are illustrated in Figures 2 and 3. All of the missense mutations map to conserved residues (Figure 2B). A single mutation in the SH2 domain was recovered (*hc134ts*), and it is the only temperature sensitive allele. Mutations found in the predicted minor lobe of the kinase domain, the region responsible for ATP binding, are *hc53* and *hc79*. Mutations found in the predicted major lobe of the kinase domain, the region responsible for recruiting target proteins to the active site, are *hc40, hc50,* and *hc108.* Because *hc50* encodes a stop codon, we performed RT-PCR on RNA from *hc50* mutants and found that the *spe-8* transcript was present (Additional file 3). Therefore, *hc50* eliminates the final 40 amino acid residues from the protein, including a small portion of the kinase domain and the entire C-terminal tail. In many protein kinases, a short C-terminal tail often contains a tyrosine residue, which can be phosphorylated, acting as a cis regulator of the active and inactive state of the protein [20]. The short C-terminus of SPE-8 does not have a tyrosine residue, so it is unclear whether this structure is autoregulatory.

A single mutation, *hc85,* originally attributed as an allele of *spe-8*[4]*,* did not have a mutation in the *spe-8* coding sequence. Our sequencing covered *spe-8* from ~ 400 bp 5’ of start through ~200 bp 3’ of stop, with no alteration from wild-type. To test whether *hc85* is actually an allele of *spe-8,* we performed a complementation test by crossing *hc85* hermaphrodites and *spe-8(hc50)* males*.* The F1 were fertile, demonstrating that *hc85* is not an allele of *spe-8.* The gene affected by *hc85* remains to be determined.

### SPE-8 localization

Using the Mos-SCI technique [21], we integrated SPE-8::GFP translational fusions into the *ttTi5605* Mos1 transposon insertion on Chromosome II. One fusion had GFP on the N-terminus, and the other had GFP on the C-terminus. Both showed green fluorescence in the male germline. We crossed worms bearing the two fusions with *spe-8(hc50)* worms to determine if either reporter fusion rescued the *spe-8* mutant phenotype. For the amino-terminal fusion, we observed 14 sterile worms out of 57 total F2, or 25%, which is expected from a monohybrid cross with no element present to rescue *spe-8*. Thus, the amino-terminal fusion did not rescue. However, for the carboxy-terminal fusion, only two of 69 F2 were sterile. This proportion, 2.9%, is significantly different from the 25% expected for no rescue (χ^2^ = 16.41; P = 0.00005), but it is similar to the percent expected if the fusion does rescue, where only 1/16 (6.2%) of the worms should be sterile. Thus, the carboxy-terminal fusion did rescue the *spe-8(hc50)* mutation, suggesting that the reporter provides an accurate portrait of SPE-8 localization. Therefore, we used the carboxy-terminal fusion to analyze SPE-8 localization.

**Figure 5:**
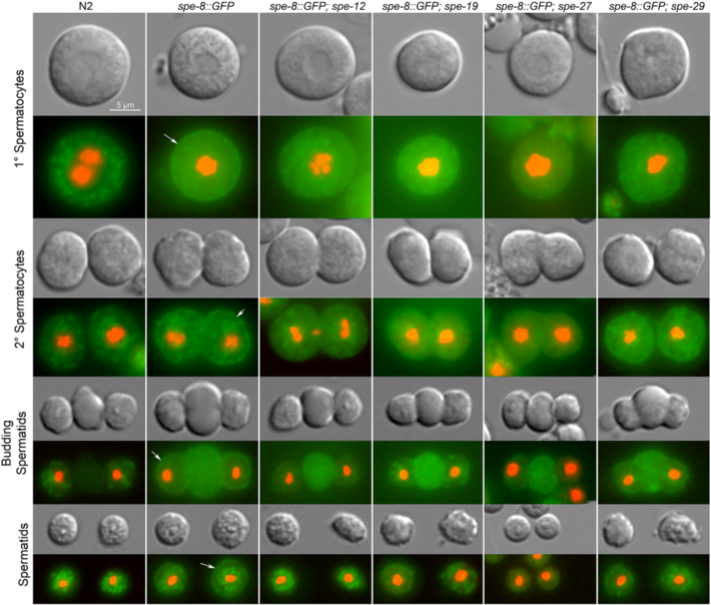
**Localization of a *spe-8::GFP *translational reporter in developing sperm.** GFP was fused to the C-terminus of the *spe-8* coding sequence and integrated into a Mos1 site on Chromosome II following the MosSCI technique. In the N2 column, cells from the unlabeled N2 strain are included as a negative control to illustrate the non-specific green autofluorescence found in *C. elegans* developing sperm. The *spe-8::GFP* column shows the green fluorescence in cells with the translational reporter. Note the arrows indicating membrane labeling not seen in the N2 controls. The remaining columns are from worms homozygous for the reporter construct insertion and a mutation in one of the *spe-8* group genes. Membrane GFP localization was absent in *spe-12(hc76), spe-19(ok3428),* and *spe-27(it132)* mutant backgrounds, but some localization was present in a *spe-29(it127)* mutant background. Red fluorescence indicates DNA labeling by Hoechst 33342.

SPE-8 localizes to the cell membrane during spermatogenesis. All cellular stages of spermatogenesis showed green fluorescence associated with the cell membrane (Figure 5, *spe-8::GFP* column). Unfortunately, developing sperm cells have autofluorescent internal structures. In comparison, control (non-reporter) developing sperm do not have green fluorescence associated with the cell membrane (Figure 5, N2 column). The primary spermatocytes expressing the reporter appear much more fluorescent even interior to the cell membrane than N2 controls, suggesting that SPE-8 may be accumulating in the cytosol and translocating to the membrane at this stage. By the secondary spermatocyte stage, the cytosol appears no more fluorescent than controls, but the plasma membrane is strongly fluorescent as the reporter has taken up residence there. At the budding spermatid stage a strong GFP signal comes from the residual body, where non-essential cellular components are disposed [22]. It is unclear if native SPE-8 enters the residual body, and an alternative possibility is that some of the fusion has degraded and segregated to the residual body. Finally, the membrane localization of GFP is most pronounced at the spermatid stage, when the cells await the activation signal.

Our results also show that SPE-8 localization is dependent upon the other *spe-8* group proteins. Localization is disrupted in all *spe-8* group mutant backgrounds with the possible exception of *spe-29* (Figure 5). In *spe-12(hc76), spe-19(ok3428),* and *spe-27(it132)* mutant backgrounds, there is no indication of reporter localization to the membrane. Indeed, the fluorescence seems to concentrate in the residual body, which might be indicative of degradation due to the loss of components of the mature activation complex. In a *spe-29(it127)* mutant background, reporter localization to the membrane is visible, but it is not as intense as in an otherwise wild-type background, perhaps as a consequence of the likelihood that the *spe-29(it127)* mutation does not result in a complete loss of function [6].

**Figure 6:**
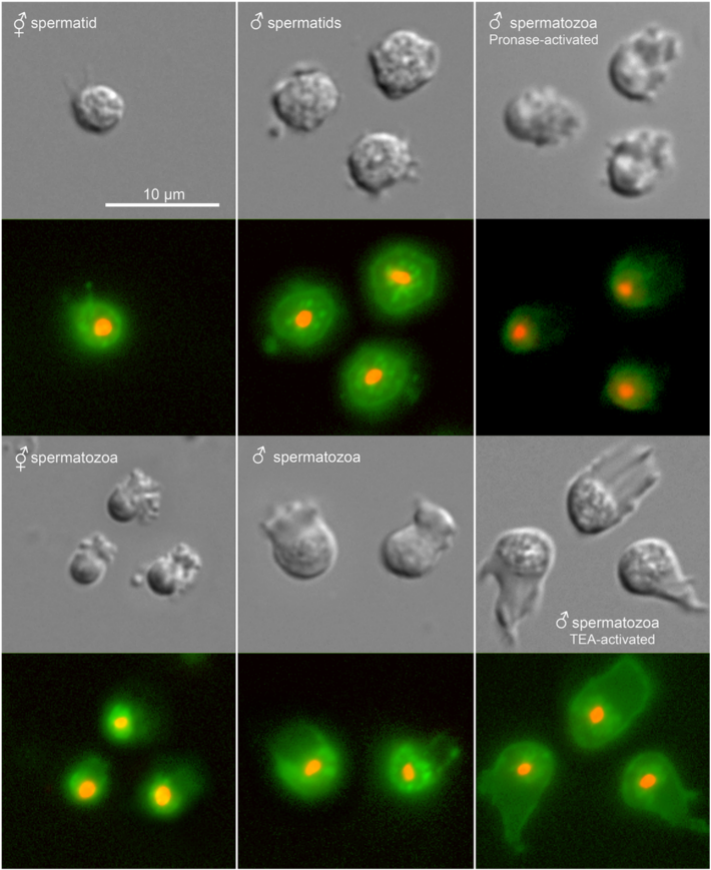
**Localization of the *spe-8::GFP *translational reporter in activated spermatozoa.** Spermatids and spermatozoa were isolated from young adult hermaphrodites (first column). The GFP fluorescence has moved to the cytoplasm in the activated spermatozoa. A similar pattern was seen in spermatids and spermatozoa isolated from young adult males (second column). Spermatozoa activated with Pronase also have cytoplasmic GFP localization (third column, top), but when activated with triethanolamine (TEA), the GFP remains at the membrane (third column, bottom).

We also investigated SPE-8 localization in activated spermatozoa. In naturally activated spermatozoa from both males and hermaphrodites, the membrane localization found in spermatids disperses into the cytosol (Figure 6). Hermaphrodite self sperm activate exclusively through the SPE-8 group pathway signaled by extracellular zinc [2], so entry of SPE-8 into the cytoplasm is a normal consequence of zinc activation through the SPE-8 group. Male sperm utilize both the SPE-8 pathway and TRY-5 pathway [3]. While activated male sperm showed the same entry of SPE-8 into the cytoplasm, it is not clear whether TRY-5 alone releases SPE-8 from the membrane. What is clear is that TRY-5 activation is completely independent of the SPE-8 group: male *spe-8* group mutants are fertile via the TRY-5 pathway, even though our results show that SPE-8 is mislocalized or absent in their sperm. SPE-8 entry into the cytoplasm also occurs in sperm activated proteolytically via Pronase *in vitro* (Figure 6). Pronase activation likely acts, at least in part, through the zinc-signaled SPE-8 group proteins, because *spe-8* group mutant spermatids cannot be activated completely to spermatozoa by exposure to Pronase [23]. Interestingly, *in vitro* activation via triethanolamine (TEA) leaves SPE-8 localized to the membrane (Figure 6), which is not surprising given that TEA bypasses the activation pathway by mimicking a downstream spike in cytosolic pH [23].

## Discussion

Here, we describe the identification, structure, and function of *spe-8,* and show that it is localized to the spermatid cell membrane but enters the cytoplasm during activation. spe*-8* was the first of the five genes of the *spe-8* group (*spe-8, -12, -19, -27,* and *-29*) to be identified by mutation. In contrast to the other four genes, whose sequences reveal little about their activity, the SPE-8 sequence suggests it belongs to one of the most prominent families of signaling molecules, the protein tyrosine kinases (PTKs) [24]. These proteins route information through cells by phosphorylating specific tyrosine residues on their substrate proteins, thereby altering their substrates' chemical activities and/or binding properties. The Src homology 2 (SH2) domain, a compact domain that recognizes and binds to specific phosphotyrosine-containing proteins, is a common protein module that is often associated with PTKs and is present in SPE-8. Beyond the strong sequence similarity to these two domains exhibited by SPE-8, the fact that all six of the identified mutations lie in conserved residues of the SH2 or PTK domain underscores the importance of these domains in SPE-8 function.

PTKs belong to two general classes, the receptor tyrosine kinases (RTKs) and the non-receptor tyrosine kinases (NRTKs) [6]. Whereas RTKs are typically bound to the plasma membrane through their own N-terminal structures, NRTKs may associate with membranes through associations of their N-terminal domains with co-receptor proteins. Based on overall sequence similarity, SPE-8 is most closely related to the Fer and Fes family of NRTKs [15]. Human Fes has both oncogenic and tumor suppressor roles, depending upon the type of tumor [14,25], and Fer is widely expressed and activated by growth factors, primarily platelet derived growth factor (PDGF) [26]. Fer is involved in many processes including cell proliferation [27,28], cell adhesion [29], cell migration [30], and even oocyte maturation [31]. Fer is also implicated in several cancers [32-34]. Interestingly, a testis-specific alternatively-spliced isoform of Fer, FerT, has a distinct, shorter N-terminus than the full-length Fer protein and participates in acrosome formation through phosphorylation of actin remodeling proteins [35].

Both Fer and Fes have, in addition to their SH2 and kinase domains, several coiled-coil domains in their N-termini, which appear responsible for activation by oligomerization and subsequent trans-autophosphorylation of these proteins [18,24]. NRTKs in general are found to localize in variable fashions either in the cytosol or bound to interior membranes via protein complexes [20]. The SPE-8 N-terminus is unique compared to all other members of the sperm expressed NRTK family in *C. elegans.* It is unclear if the N-terminus of SPE-8 is responsible for formation of dimers or oligomers, or if autophosphorylation occurs. However, we speculate that this structure may be responsible for protein-protein interactions, especially given its proline-rich region, many of which are implicated in protein-protein docking or in binding SH3 domains [20,36,37]. Perhaps the failure of our N-terminal GFP fusion to rescue the *spe-8(hc50)* mutation reflects the importance of the N-terminus in SPE-8 function. Unlike the unique N-terminus, the SPE-8 C-terminus matches those of some of the family members.

Whatever the function of the C- and N-terminal domains, it is clear that interactions among the SPE-8 group proteins act to localize or stabilize SPE-8 at the plasma membrane. Localization was disrupted in mutant backgrounds for *spe-12, spe-19,* and *spe-27,* and perhaps for *spe-29.* It is not surprising that SPE-12 and SPE-19 proteins are required for proper SPE-8 localization because both have transmembrane domains, and SPE-12 has been shown experimentally to reside in the cell membrane [5,7]. However, the requirement of SPE-27 for proper SPE-8 localization is unexpected, given that SPE-27 is predicted to be cytosolic [38]. These results suggest SPE-27 may also be located at the plasma membrane. For SPE-29, the results were not clear. SPE-8 appeared to localize to the membrane in a mutant *spe-29* background, but seemingly not as intensely as in a wild-type genomic background.

While SPE-8 associates with the membrane in developing sperm, its localization changes once the cells activate to spermatozoa. The protein appears to leave the membrane and become cytoplasmic in natively activated spermatozoa originating from both males and hermaphrodites. In both cases, the sperm are exposed to extracellular zinc, which acts through the SPE-8 group proteins to induce activation [2]. We have no method for observing SPE-8::GFP localization in sperm activated only through the TRY-5 pathway. It is not possible to activate sperm *in vitro* with TRY-5, and *spe-8* group mutant male sperm are activated only by TRY-5, but SPE-8::GFP is mislocalized due to the *spe-8* group mutation. In any case, SPE-8 is dispensable for TRY-5 activation. Not only does TRY-5 activation occur in *spe-8* mutant backgrounds, but SPE-8 is also mislocalized in *spe-12, spe-19,* and *spe-27* mutant backgrounds.

It is tempting to speculate as to the phosphorylation target of SPE-8. One possible target is SPE-6. SPE-6 has a predicted role maintaining the spermatid state by inhibiting activation until the SPE-8 group signal is transduced into the cell [39]. One hypothesis is that SPE-8 downregulates SPE-6 through tyrosine phosphorylation. SPE-6 protein exists in a perinuclear halo in spermatids (Jackson Peterson and Diane Shakes, personal communication), so the translocation of SPE-8 from the plasmalemma into the cytosol might enable interaction between the two proteins. Indeed, the NetPhos 2.0 Server [40] identifies four of the tyrosine residues in SPE-6 as high probability candidates for phosphorylation (data not shown). Another possible target is a *C. elegans* homolog of MPOP, the major sperm protein polymerization organizing protein identified in *Ascaris suum*[41,42], although a *C. elegans* homolog has yet to be found. MPOP is an integral membrane protein distributed throughout the plasmalemma, but it is phosphorylated by a tyrosine kinase only on the leading edge of the pseudopod, where it nucleates MSP assembly to drive pseudopodial motility. However, SPE-8 is not likely the tyrosine kinase responsible for a putative *C. elegans* MPOP phosphorylation because SPE-8 appears restricted to the cell body in active spermatozoa (Figure 6).

SPE-8 shares both primary sequence and protein domain architecture similarity with at least 33 other predicted paralogs in *C. elegans*, 29 of which have been shown to be upregulated during spermatogenesis in at least one of two studies. We include genes identified as sperm-expressed in either study because each study has slight limitations: (i) the microarray studies [9,10] did not include all genes in the genome and may suffer from cross-hybridization, and (ii) the RNA deep sequencing study [16] may miss early acting sperm genes expressed whose transcripts are degraded prior to cellular encapsulation at the primary spermatocyte stage. While the majority of these genes have not received experimental attention, 30 have knockout alleles available. Only four of those knockouts produced a sterile phenotype, with the rest having no phenotype (Additional file 2). RNAi reports suggest that some of the paralogs have somatic function, although even *spe-8* has an RNAi phenotype of hypersensitivity to hypoxia [43]. Whether or not these paralogs have significant function in sperm remains unclear, but the fact that so few have phenotypes for the knockout alleles suggests that they have redundant function. The fact that so many kinases appear upregulated in sperm indicates that protein phosphorylation is a prominent regulatory mechanism during spermatogenesis. Such a conclusion is not surprising since (i) post-translational modification is the major regulatory mechanism available, owing to the cessation of gene expression at the spermatid stage, and (ii) protein phosphorylation is rapid, befitting the swift morphological transformations that take place during spermiogenesis.

Extracellular zinc is the signal for sperm activation transduced through the SPE-8 complex [2]. The zinc signal is transmitted across the membrane to SPE-8 tethered just inside the membrane. It is unclear how reception of the zinc signal releases SPE-8 from the membrane. Zinc is an important signaling molecule in numerous cell types. For instance, the activity of most cell types in the mammalian immune system is regulated by zinc [44]. Zinc alters sperm activity across many species, but its effect can be either positive or negative, depending upon the species (reviewed in [2]). Perhaps a more relevant example is the activation of p70 S6 kinase by extracellular zinc signaling in the progression of the cell cycle, with the possible involvement of intermediate NRTKs [45]. While the identities of all intermediate factors and their specific interactions are not known, extracellular zinc induced signaling involving kinases is widespread, and our work on SPE-8 helps in understanding how spatial localization is involved in the signaling events.

## Conclusions

The spermatid activation signal initiated by extracellular zinc is transduced into the cell via the SPE-8 group proteins. Of the five proteins known to compose this pathway, only SPE-8 has a predicted functional domain, that being a non-receptor protein-tyrosine kinase. Three have predicted membrane domains (SPE-12, SPE-19, and SPE-29), and SPE-27 has no predicted domains. The association of the SPE-8 group proteins has been unclear. Our results suggest that the SPE-8 group proteins form a functional complex localized at the plasma membrane, and that SPE-8 is correctly positioned only when all members of the SPE-8 group are present, with the possible exception of SPE-29. Further, SPE-8 is released from the membrane when the activation signal is transduced into the spermatid. The identity of its phosphorylation target(s) is not yet clear, nor is whether SPE-8 kinase activity is up- or down-regulated by the activation signal. SPE-8 is also a member of a large family of sperm-expressed non-receptor protein tyrosine kinases in *C. elegans*, suggesting that the rapid changes associated with spermatogenesis – and spermatid activation in particular – are brought about, at least in part, through changes in phosphorylation.

## Methods

### Strains and general nematode methods

Worm strains were cultured by standard methods [46]. Some strains were provided by the Caenorhabditis Genetics Center (CGC), which is funded by NIH Office of Research Infrastructure Programs (P40 OD010440). Others were either generated during the present work or were graciously provided by Samuel Ward and Steven W. L’Hernault. The following strains harbored *spe-8* group gene mutations: BA785: *spe-8(hc40) I*, BA784: *spe-8(hc50) I*, BA786: *spe-8(hc53) I*, BA788: *spe-8(hc79) I*, BA801: *spe-8(hc85) I* (although we show here that *hc85* is not an allele of *spe-8*), BA787: *spe-8(hc108) I*, BA797: *spe-8(hc134ts) I*, VC2971: *spe-19(ok3428)/unc-51(e369) rol-9(sc148) V,* BA963: *spe-27(it132ts) IV,* BA959: *spe-29(it127) dpy-20(e1282) IV,* BA783: *spe-12(hc76) I.* Translational GFP reporters were integrated into the *ttTi5605* Mos1 insertion site in the strain EG4322: *ttTi5605 II; unc-119(ed9) III.* Sperm were activated *in vitro* using Pronase and triethanolamine following standard protocols [23].

### Differential PCR analysis of *spe-8*

We used PCR to assess sperm-specific expression of *spe-8.* PCR was conducted on cDNA libraries constructed from either *fem-1(hc17ts)* or *fem-3(q23ts)* mutants (kindly provided by Harold Smith). Three different products were amplified using different primer pairs: (i) a *spe-8* product (primers F53G12.6Lb and F53G12.6 L3'), (ii) a *spe-12* product as a sperm upregulated positive control [7], and (iii) a product from F2SH8.1, which is expressed somatically (Jeremy Nance, personal communication). PCR primer sequences are listed in Additional file 1. PCR reaction conditions and cycling followed standard protocols [47].

### Microinjection transformation rescue

We amplified a 4,273 bp fragment from wild-type worms containing the entire F53G12.6 gene, including 919 base pairs 5' of the start codon, and 592 base pairs 3' from the stop codon. We used Phusion High Fidelity DNA Polymerase (Thermo Scientific) for amplification following manufacturer’s protocols. For transformation rescue, a mixture containing 10 ng/μl of this PCR fragment, along with 100 ng/μl of the pCFJ104 plasmid containing the *Pmyo-3::mCherry::unc-54* transcriptional fusion to track transformed progeny. The DNA mix was injected into young adult *spe-8(hc50)* mutant hermaphrodites that were then paired with males of the same mutant genotype. F1 and F2 hermaphrodite transformants were scored for self-fertility at 25°C using non-transformant sibs as controls.

### Sequencing the *spe-8* mutants

To sequence the *spe-8* mutant alleles, the *spe-8* genomic region from the mutants was amplified by PCR either in two ~1,300 bp fragments (for mutations *hc40, hc50, hc53, hc79, hc108,* and *hc134ts*)*,* or in a single large product corresponding to the rescuing fragment (for *hc85*). Phusion High Fidelity DNA Polymerase was used in amplification, and the fragments were sequenced by the Sanger method using the PCR primers and primers internal to the two fragments. All primer sequences are listed in Additional file 1.

### RT-PCR

RNA was extracted from a mixed age population of *spe-8(hc50)* worms. The worms were rinsed 4 times with M9 buffer, disrupted with a homogenizer in lysis buffer from the GeneJET™ RNA purification kit (Thermo Scientific), and the RNA was extracted following the manufacturer’s protocols. The RNA samples were treated with RQ1 DNase (Promega), and reverse transcription was performed with Maxima™ Reverse Transcriptase (Thermo Scientific) per manufacturer’s instructions with oligo (dT). We then multiplexed *spe-8* specific primers (F53G12.6-F5 and F53G12.6-R5; see Additional file 1) with primers specific to *act-2,* the *C. elegans* ortholog of β-actin (see Additional file 1 for primer sequences) for amplification using Phusion High Fidelity DNA Polymerase (Thermo Scientific) per manufacturer’s protocols.

### SPE-8 localization

Two translational GFP fusions with *spe-8* were constructed following the Mos-SCI technique [21]. Both constructs included 838 bp of the sequence upstream of the start codon and 1039 bp downstream of the stop codon. The GFP coding sequence from plasmid pPD95.75 (Fire Lab *C. elegans* vector kit obtained from Addgene) was inserted either at the 5’ or the 3’ end of the genomic *spe-8* sequence. The stop codon was eliminated from (i) GFP for the amino-terminal construct, or (ii) s*pe-8* for the carboxy-terminal construct. The various segments of the construct were amplified by PCR using Phusion High Fidelity DNA Polymerase (Thermo Scientific), and they were combined following the PCR fusion technique described by Hobert [48]. Primers used in the construction are listed in Additional file 1. The full-length constructs were cloned into the vector pCFJ151, which targets the *ttTi5605* Mos1 transposon insertion on Chromosome II for homologous recombination. Following the Mos-SCI protocol [21], we recovered a homozygous integrated copy of each construct. The integrated reporters were crossed with *spe-8(hc50)* to determine if the reporters rescued *spe-8* mutant sterility. The C-terminal reporter (*zqIs8[spe-8::GFP]*) rescued *spe-8* mutant sterility (see Results) and was used in localization studies. We crossed the *zqIs8[spe-8::GFP]* translational reporter with mutations in the various *spe-8* group genes to obtain strains that were homozygous for both *zqIs8* and for the *spe-8* group mutations. Microscopy was performed with a Nikon Eclipse Ti-S inverted microscope fitted for Differential Interference Contrast microscopy and epifluorescence. Localization was examined in the following strains: ZQ125: *zqIs8 II; unc-119(ed3) III,* ZQ126: *spe-12(hc76) I; zqIs8 II,* ZQ127: *zqIs8 II; spe-29(it127) dpy-20(e1282ts) IV,* ZQ128: *zqIs8 II; spe-19(eb52) V,* and ZQ137: *zqIs8 II; spe-27(it132ts) IV.*

## Competing interests

The author(s) declare that they have no competing interests.

## Authors’ contributions

PJM conceived of the *spe-8* identification study and its design, carried out the discovery of the *spe-8* coding sequence, and aided in the sequencing of the mutants, discovery of paralogs, and drafting of the manuscript. JNC participated in the sequencing of the mutants and drafting of the manuscript. UN contributed to the construction of the reporter strains, RT-PCR, and drafting the manuscript. NGS modeled the SPE-8 protein and participated in drafting the manuscript. CWL conceived and designed the localization studies, aided in project design, created the reporter constructs, performed the microscopy, and contributed to drafting the manuscript. All authors read and approved the final manuscript.

## Supplementary Material

Additional file 1 Primers used in PCR amplification and sequencing. Click here for file

Additional file 2 ***C. elegans *** **SPE-8 paralogs.** SPE-8 and its paralogs with a match significance ≤10^-30^. The chromosome and start position are given for each paralog, as is the RPKM (Reads Per Kilobase of exon model per Million mapped reads) from RNA-seq for sperm gene identification [16]. The presence of a value in RPKM indicates upregulation in sperm. *fem-3/fem-1* expression ratio, where larger ratios indicate upregulation in sperm [9]. If available, the identity of a knockout allele, and its phenotype are listed. Phenotype data were taken from http://www.wormbase.org**, version WS240.** Click here for file

Additional file 3 **RT-PCR for *spe-8* in a *spe-8(hc50) *** **mutant.** RNA was extracted and DNase treated and then subjected to RT-PCR for both a fragment of the *spe-8* coding sequence and for a fragment of the gene *act-2*, a homolog of β-actin. The primers for *spe-8* and *act-2* are listed in Additional file 1. Both primer sets cross introns, and the resulting products would be larger if amplified from genomic DNA (*act-2*: 1050 bp; *spe-8*: 565 bp). Click here for file
