# Supplementary material for: SPE-8, a protein-tyrosine kinase, localizes to the spermatid cell membrane through interaction with other members of the SPE-8 group spermatid activation signaling pathway in C. elegans
Source: BMC Genet. 2014 Jul 14;15:83. doi: 10.1186/1471-2156-15-83 (PMC4105102; doi:10.1186/1471-2156-15-83)
Supplement: Additional file 1 — Primers used in PCR amplification and sequencing. [file 1471-2156-15-83-S1.docx]

### Additional File 1: Primers used in PCR amplification and sequencing.

| Primers | Sequence (5' to 3') | 5' end location | Comment |
| --- | --- | --- | --- |
| Prom-F | AAGGAGAAATGGGTCCACAAGG | 117,715 | For PCR amplifying entire spe·8 gene for transformation rescue and sequencing (4,273 bp PCR product) |
| 3’ UTR-R | GATTTTCCGAACGCCAATTGCCG | 121,035 |  |
| F53G12.6L5' (f) | GAAACTGTGTTAGCGCCACTT | 118,321 | For amplifying left spe·8 genomic fragment for sequencing template (1352 bp PCR product) |
| F53G12.6L3' (r) | CAACGTCATGCCCTCATAAAT | 119,672 |  |
| F53G12.6R5' (f) | GTACGCCTTCAAATCCATCTG | 119,594 | For amplifying right spe-8 genomic fragment for sequencing template (1356 bp PCR product) |
| F53G12.6R3' (r) | TCTGTTTGTGTGCGTTGTGTG | 120,949 |  |
| F53G12.6-F1  F53G12.6-F2  F53G12.6-F2a  F53G12.6-F3  F53G12.6-F4  F53G12.6-F5  F53G12.6-F5a  F53G12.6-F6 | GTAAGTTGCTTGTTCGCTCATTCTGA  TACTCCAATTCCAGAAACCC  CGCCTATGTGATTAGTATCAA  TCCCTGAAGCATTATGCGG  TACGCCTTCAAATCCATCTGTGAGC  GCCTGCGGTATTGATTATTTGCACGG  GGATTGTCGGTCAAAGGAGTT  TAGAGCTACCAGTGTTCATTGCC | 117,969  118,539  118,887  119,065  119,595  120,099  120,260  120,610 | Forward internal sequencing primers |
| F53G12.6-R1  F53G12.6-R1a  F53G12.6-R2  F53G12.6-R3  F53G12.6-R4  F53G12.6-R4a  F53G12.6-R5  F53G12.6-R6 | TTGGGGTCTTGGCAAGATTGTCC  TCCGCATAATGCTTCAGGGAG  TTTGGCAAATTCGGCAAATCGGC  AACGTCATGCCCTCATAAATCGG  ACAATTTCTCGCCGCAATATCC  TGTACTTGACCGGCAGACATC  TCTCGTACTGCCTGCTTTGCTCG  ACAGTCAAATATTTTCAGGGGTGGGG | 118,604  119,085  119,163  119,672  120,165  120,324  120,664  120,914 | Reverse internal sequencing primers |
| Vector-F | AACGACGGCCAGTGAATTGTAATAC | - | For amplification of 5’ *spe-8* sequence from the *fem-3(q23ts)* cDNA library |
| F25H8.1-F  F25H8.1-R | TTGCTTTGGACATGTCATTTGACG  CAATTGAGCATTCTTCCGTTCTGG | 9,931,711  9,931,092 | For amplification of a 526bp fragment of the F25H8.1 cDNA |
| *spe-12*-F  *spe-12*-R | GTTAAATGCGGAGCACAGCGAG  TCAATCGAGATCTTCTACAGTTCG | 8,226,206  8,224,608 | For amplification of a 715 bp fragment of the *spe-12* cDNA |
| GFP-F  GFP-R | CAGCTATGACCATGATTACGC  CAGACAAGTTGGTAATGGTAGC | -  - | Outer primers to amplify GFP plus flanking sequence from pPD95.75 |
| *spe-8* prom-F | gtgtCCTAGGcgggagatcaaggtaatccg | 117,271 | For amplification of the *spe-8* promoter (lower case on 3’ end), plus an AvrII restriction site (uppercase) and additional bases efficient digest (lowercase on 5’ end) |
| *spe-8* 3’UTR-R | gtgtGCGCGCttcttccagttccagttctgttc | 121,919 | For amplification of the *spe-8* 3’ UTR (lower case on 3’ end), plus a BssHII restriction site (uppercase) and additional bases efficient digest (lowercase on 5’ end) |
| *spe-8-GFP_B* | gaaaagttcttctcctttactcatCACTGCCACCGCCGCGGGTTTTC | 120,867 | With *spe-*8 prom-F for amplification of promoter plus coding sequence with region that overlaps GFP start (lowercase) |
| *spe-8-GFP_C* | gcatggatgaactatacaaatagTAAATATCTGAAAATCCCCCCAC | 120,871 | With *spe-8* 3’UTR-R for amplification of 3’UTR with region that overlaps GFP stop (lowercase) |
| *GFP-spe-8_B* | gtgaaaagttcttctcctttactcatTTGTTGTTTGTTAAAAACTATTAAATGAATG | 118,108 | With *spe-*8 prom-F for amplification of promoter with region that overlaps GFP start (lowercase) |
| *GFP-spe-8_C* | catggcatggatgaactatacaaaATGCGATCAAAAAGTTCGGAAGGT | 118,109 | With *spe-8* 3’UTR-R for amplification of coding sequence plus 3’UTR with region that overlaps GFP stop (lowercase) |
| actinF  actinR | GTATGGGACAGAAAGACTCG  CGTCGTATTCTTGCTTGGAG | 11,077,988  11,076,938 | For RT-PCR amplification of a fragment of *act-2*, the *C. elegans* ortholog of β-actin |
