# Supplementary material for: SPE-8, a protein-tyrosine kinase, localizes to the spermatid cell membrane through interaction with other members of the SPE-8 group spermatid activation signaling pathway in C. elegans
Source: BMC Genet. 2014 Jul 14;15:83. doi: 10.1186/1471-2156-15-83 (PMC4105102; doi:10.1186/1471-2156-15-83)
Supplement: Additional file 2 — C. elegans SPE-8 paralogs. SPE-8 and its paralogs with a match significance ≤10-30. The chromosome and start position are given for each paralog, as is the RPKM (Reads Per Kilobase of exon model per Million mapped reads) from RNA-seq for sperm gene identification [16]. The presence of a value in RPKM indicates upregulation in sperm. fem-3/fem-1 expression ratio, where larger ratios indicate upregulation in sperm [9]. If available, the identity of a knockout allele, and its phenotype are listed. Phenotype data were taken from http://www.wormbase.org, version WS240. [file 1471-2156-15-83-S2.docx]

### Additional File 2. *C. elegans* SPE-8 paralogs

SPE-8 and its paralogs with a match significance ≤10^-30^. The chromosome and start position are given for each paralog, as is the RPKM (Reads Per Kilobase of exon model per Million mapped reads) from RNA-seq for sperm gene identification [14]. The presence of a value in RPKM indicates upregulation in sperm. *fem-3/fem-1* expression ratio, where larger ratios indicate upregulation in sperm [9]. If available, the identity of a knockout allele, and its phenotype are listed. Phenotype data were taken from [www.wormbase.org](http://www.wormbase.org), version WS240.

| GeneName | Chr | Start Location | RPKM | *fem-3/ fem-1* | KO allele available | KO allele Phenotype | RNAi Phenotype |
| --- | --- | --- | --- | --- | --- | --- | --- |
| F26E4.5 | I | 9771196 | 0.112 | 11.8 | *gk119443*^1^ | - | Neuronal |
| F23C8.7 | I | 2425237 | 0.060 | 10.4 | *gk462577*^1^ | - | aldicarb^R^ |
| C25A8.5 | IV | 6999220 | 0.165 | 21.2 | *gk204642*^1^ | - | aldicarb^R^ |
| *kin-26* | IV | 7877926 | -- | 18.8 | *gk119443*^1^ | - | - |
| *kin-5* | IV | 9969307 | 0.170 | 20.6 | *tm4072*^2^ | viable | - |
| *frk-1* | IV | 10043372 | -- | 15.9 | *ok760*^2^ | - | embryonic |
| T25B9.4 | IV | 10757835 | 0.080 | 19.2 | *tm4141*^2^ | viable | - |
| C55C3.4 | IV | 5678259 | 0.181 | 21.8 | - | - | - |
| C35E7.10a | I | 10812998 | 0.109 | NA | *gk1104*^2^ | sterile | - |
| ZK622.1 | II | 5293454 | 0.104 | 18.9 | *tm4256*^2^ | viable | - |
| T06C10.3 | IV | 7508502 | 0.084 | 19.6 | *tm4262*^2^ | viable | aldicarb^R^ |
| W01B6.5 | IV | 10075860 | 0.076 | 10.6 | *gk210136*^1^ | - | - |
| F57B9.8 | III | 6935428 | 0.056 | 19.4 | *tm4451*^2^ | sterile | numerous somatic |
| *kin-31* | III | 8671319 | 0.079 | 6.4 | *tm323*^2^ | viable | - |
| *kin-14* | I | 7079695 | 0.031 | 6.0 | *WBVar00091162*^2^ | - | small |
| F22B3.8 | IV | 11425403 | 0.031 | 10.8 | *tm4534^2^* | viable | growth |
| ZK593.9 | IV | 10940534 | -- | 8.5 | *gk663211*^1^ | - | - |
| *abl-1* | X | 10625286 | -- | 0.5 | *tm4301*^2^ | viable | numerous somatic |
| F59A3.8 | I | 5525516 | 0.076 | 9.1 | *tm5118*^2^ | viable | - |
| R11E3.1 | IV | 4789285 | 0.176 | 30.0 | *tm4276*^2^ | viable | - |
| ZC581.7 | I | 6662426 | 0.174 | 19.5 | *tm4166*^2^ | viable | - |
| Y116A8C.38b | IV | 17130344 | 0.065 | 25.7 | *tm4106*^2^ | viable | - |
| Y116A8C.24a | IV | 17070989 | 0.054 | 22.8 | - | - | - |
| R05H5.4 | II | 10199855 | 0.128 | 3.7 | *ok2875*^2^,  *tm4354*^2^ | -  viable | - |
| T25B9.5 | IV | 10754718 | -- | 10.2 | *tm4060*^2^ | sterile | aldicarb^R^ |
| Y69E1A.3 | IV | 10951893 | -- | 14.3 | *tm4327*^2^ | sterile | aldicarb^R^ |
| *src-2* | I | 14320892 | -- | 0.7 | *ok819*^2^  *tm1139*^2^ | -  viable | P-vul, lethal |
| C18H7.4 | IV | 592954 | 0.156 | 15.2 | *tm3902*^2^ | viable | - |
| *kin-24* | IV | 9841189 | 0.054 | 10.4 | - | - | - |
| *src-1* | I | 1564278 | -- | NA | *ok2685*^3^ | movement defects | numerous somatic |
| *kin-21* | IV | 9832557 | 0.052 | 7.3 | *gk3184*^2^ | viable | endocytosis |
| Y52D5A.2 | IV | 5464381 | -- | NA | *tm4125*^2^ | - | aldicarb^R^ |
| K09B11.5b | IV | 13434237 | -- | 10.6 | *tm4444*^2^ | viable | - |

**^1^**Indicates nonsense allele; ^2^indicates an indel; ^3^lesion not known.

NA in *fem-3/fem-1* column indicates genes not included in the microarray experiment.
