# Supplementary material for: SPE-8, a protein-tyrosine kinase, localizes to the spermatid cell membrane through interaction with other members of the SPE-8 group spermatid activation signaling pathway in C. elegans
Source: BMC Genet. 2014 Jul 14;15:83. doi: 10.1186/1471-2156-15-83 (PMC4105102; doi:10.1186/1471-2156-15-83)

### Additional File 3: RT-PCR for *spe-8* in a *spe-8(hc50)* mutant

RNA was extracted and DNase treated and then subjected to RT-PCR for both a fragment of the *spe-8* coding sequence and for a fragment of the gene *act-2*, a homolog of β-actin. The primers for *spe-8* and *act-2* are listed in Additional File 3. Both primer sets cross introns, and the resulting products would be larger if amplified from genomic DNA (*act-2*: 1050 bp; *spe-8*: 565 bp).


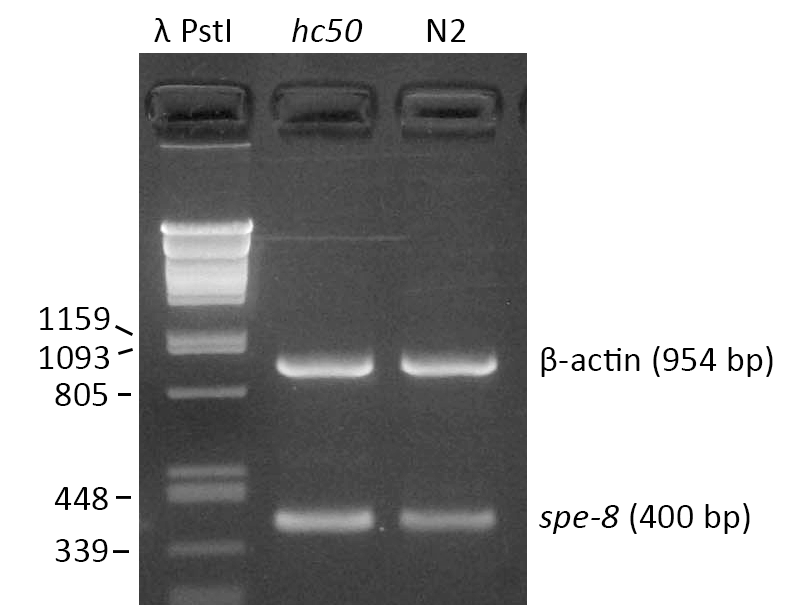

Supplement: Additional file 3 — RT-PCR for spe-8 in a spe-8(hc50) mutant. RNA was extracted and DNase treated and then subjected to RT-PCR for both a fragment of the spe-8 coding sequence and for a fragment of the gene act-2, a homolog of β-actin. The primers for spe-8 and act-2 are listed in Additional file 1. Both primer sets cross introns, and the resulting products would be larger if amplified from genomic DNA (act-2: 1050 bp; spe-8: 565 bp). [file 1471-2156-15-83-S3.docx]
